# Enhanced cognitive behaviour therapy for adolescents with eating disorders: development, effectiveness, and future challenges

**DOI:** 10.1186/s13030-024-00315-7

**Published:** 2024-09-04

**Authors:** Riccardo Dalle Grave, Simona Calugi

**Affiliations:** Department of Eating and Weight Disorders, Villa Garda Hospital, Via Monte Baldo, 89 37016 Garda, VR Italy

**Keywords:** Anorexia nervosa, Bulimia nervosa, Eating disorders, Treatment, Cognitive behaviour therapy, Family-based treatment, Outcome, Adolescents

## Abstract

Eating disorders can significantly impact the psychosocial functioning and physical health of adolescents. Early and effective treatment is crucial to prevent long-lasting and potentially devastating adverse effects. The National Institute for Health and Care Excellence has recommended cognitive behaviour therapy (CBT) for eating disorders in adolescents when family therapy is unacceptable, contraindicated, or ineffective. This recommendation was primarily based on the review of promising results from the enhanced version of CBT (CBT-E) adapted for adolescents with eating disorders aged between 12 and 19 years. A non-randomized effectiveness trial has also shown that CBT-E achieved a similar outcome to family-based treatment (FBT) at 6- and 12-months. CBT-E has several advantages. It is acceptable to young people, and its collaborative nature suits ambivalent young patients who may be particularly concerned about control issues. The transdiagnostic scope of the treatment is an advantage as it can treat the full range of disorders that occur in adolescent patients. It is an individual one-on-one treatment that does not necessitate the full involvement of the family. This approach is particularly beneficial for families that can only provide limited support. Future challenges include clarifying the relative efficacy of CBT-E and family therapy for the treatment of adolescent patients with eating disorders in a randomized control trial and increasing its effectiveness, identifying the reasons for the lack of response, and modifying the treatment accordingly.

## Background

Adolescents with eating disorders experience significant impacts on their psychosocial functioning and physical health. Early and effective treatment is essential to prevent enduring and potentially severe negative effects [1]. The National Institute for Health and Care Excellence (NICE) recommends cognitive behaviour therapy (CBT) for eating disorders in adolescents when family therapy is unacceptable, contraindicated, or ineffective [2]. This recommendation is largely based on the review of promising outcomes from the enhanced version of CBT (CBT-E) adapted for adolescents aged 12 to 19 years [3].

In this article, we describe the rationale for using CBT-E with adolescents, detail the main adaptations of the treatment for this age group, provide a general overview of the therapy, present data on its effectiveness, and discuss future challenges that need to be addressed.

## Rationale to adapt CBT-E for adolescents

Enhanced Cognitive Behavior Therapy (CBT-E) is a specialized form of CBT developed at the Centre for Research on Eating Disorders at Oxford (CREDO) designed to augment the effectiveness of the original CBT for bulimia nervosa and to address the cognitive and behavioural processes that maintain the psychopathology of eating disorders rather than focusing on specific diagnostic categories such as anorexia nervosa, bulimia nervosa, binge-eating disorder, and other eating disorders [4, 5].

Initially designed for adult patients with eating disorders manageable on an outpatient basis [4, 5], CBT efficacy has been demonstrated in numerous clinical trials [6] and is recommended by the National Institute for Health and Care Excellence for all adults with eating disorders.

The idea of adapting CBT-E for adolescents was developed at the Department of Eating and Weight Disorders at Villa Garda Hospital, Italy, during a periodic supervision visit by Professor Fairburn, the acknowledged pioneer of CBT for eating disorders. Two primary clinical observations prompted us to embark on this project. Firstly, noting that young patients with eating disorders exhibit the same specific psychopathology as adults—a fact later empirically demonstrated using a network approach by [7]—we hypothesized that adolescents could benefit from CBT-E due to its focus on addressing this eating disorder psychopathology. Secondly, although young patients are often in an extreme egosyntonic phase of their disorder, they can be actively engaged in individual psychological treatment, as my colleagues and I have observed over many years of clinical practice.

In addition, CBT-E has several features that make it particularly well-suited for younger patients [8]:

- It is a psychological treatment that incorporates numerous strategies to engage the patient—an essential aspect when working with adolescents, who often feel very ambivalent about beginning treatment.
- It employs a collaborative approach to enhance the patient's overall sense of control—a relational style well-suited to younger patients for whom control, autonomy, and independence are particularly relevant themes.
- It is straightforward to understand and administer, offering a flexible and individualized approach tailored to the specific needs of young patients at various stages of their physical and cognitive development.
- The transdiagnostic nature of CBT-E allows it to address all diagnostic categories of eating disorders, making it applicable to a broad spectrum of adolescent patients who frequently overvalue control over eating per se rather than the control of shape and weight [9].
- It is an individual one-on-one treatment that does not necessitate the full involvement of the family. This approach is particularly beneficial for families that can only provide limited support.

## Main adaptations of CBT-E for adolescents

Adolescents with eating disorders often view their condition as egosyntonic and may not recognize they have a problem to address, frequently making their engagement in treatment difficult. This challenge has spurred the development of treatments based on the "disease model," which conceptualizes the illness as separate from the individual (i.e., externalization). Such a strategy allows parents or clinicians to take direct action against the eating disorder instead of actively involving the patient in the decision to address it. FBT is a prime example of this model [10]. FBT considers adolescents as unable to control their behaviour, as it is the eating disorder that controls it and, therefore, requires parents to supervise and control their child's eating habits [11].

**Fig. 1:**
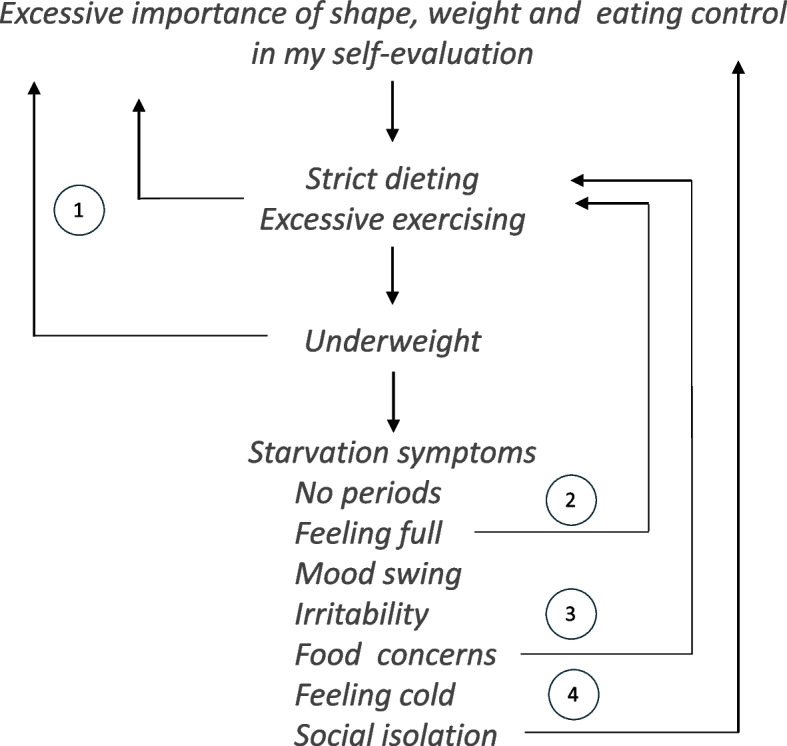
Example of personal formulation of an adolescent patient with an eating disorder highlighting how the effects of significantly low weight maintain the eating problem. 1, being underweight, strict dieting and excessive exercising are not seen as a problem but as achievements; 2, food concerns make the dieting even more extreme, rigid and difficult to modify; 3. feeling full in interpreted as having eaten too much and prompts intensification of dieting; 4, social isolation prevents experiences that can help reduce the importance attributed to shape, weight, and eating control

Conversely, CBT-E is grounded in a "psychological model," which does not separate the eating disorder from the adolescent patient. This approach highlights that young individuals can regain control by actively participating in treatment [11]. The psychological perspective underpinning CBT-E clarifies why the treatment avoids using "prescriptive" or "coercive" methods, as these can often increase resistance to change in adolescents. Step One of CBT-E does not focus on immediate weight regain. Instead, it aims to help the adolescent understand the nature of their eating problem and make a personal decision to address the change. This is achieved through a collaborative process where the therapist and patient develop a personalized formulation (Fig. 1) that includes the main maintenance processes of the eating disorder. This formulation helps the adolescent understand their eating disorder and the self-perpetuating cycles that maintain its expressions, which are primarily driven by a self-evaluation system predominantly based on controlling shape, weight, and eating.

Based on this understanding, some sessions focus on the implications of change. Creating a more effective self-evaluation system requires the adolescent to decide to address weight regain and other extreme weight-control behaviours. If the adolescent does not realize the need to tackle their problem, the treatment cannot advance and may need to be postponed, although such cases are uncommon.

Once the adolescent is engaged and consents to the change process, their eating disorder psychopathology is addressed through a flexible and personalized set of sequential cognitive and behavioural strategies and procedures integrated with progressive education. CBT-E for adolescents mirrors the adult version in its adaptability. It can be administered in a "focused" form, concentrating solely on the eating disorder psychopathology, or in a "broad" form, which also addresses one or more external maintenance processes such as clinical perfectionism, core low self-esteem, marked interpersonal difficulties, and/or mood intolerance if these are severe, seem to maintain the eating disorder psychopathology and hinder change.

Parents are involved in creating an optimal family environment to support the adolescent's change and, with the adolescent's agreement, help their child implement some treatment procedures.

Since some medical complications associated with eating disorders (e.g., osteopenia and osteoporosis, arrest or delay in growth, functional and structural changes in the brain) are particularly severe in adolescents [12], periodic medical assessments and a lower threshold for hospital admission are essential components of CBT-E for adolescents throughout the entire course of treatment.

## CBT‐E for adolescents with eating disorders: an overview

### Structure

CBT-E for adolescents involves two assessment/preparatory sessions followed by three main steps, one or more review sessions, and three post-review sessions (see Table 1). Treatment lasts about 20 weeks in patients who are not underweight. In contrast, the standard 40 weeks of adult CBT-E in underweight patients may be shortened to about 30 weeks, as adolescents tend to restore a normal body weight faster than adults [13]. Parents are required to participate in a solo interview lasting about 90 min during the first week of treatment. Following this, the patient and parents attend some to eight joint sessions of 15 to 20 min each immediately after the patient's session.

**Table 1:** The core elements of the focused CBT-E version for adolescents with eating disorders

**Assessment and preparation**
The aims are to assess the nature of the young person's eating problem and engage them in the decision to begin Step One of CBT-E
This phase involves two appointments within one week and involves
• Assess the nature and severity of eating disorder
• Describe the nature and organization of CBT-E
• Describe the nature and organization of CBT-E
• Evaluate the pros and cons to start the Step One
**Step one – Starting well and deciding to change**
The aims are to engage the patient in treatment and change, including addressing weight regain. The appointments are twice weekly for 4 weeks and involve the following:
• Jointly creating the personal formulation of the processes maintaining the eating disorder
• Establishing real-time self-monitoring
• Educating about body weight regulation and fluctuations, the adverse effects of dieting
• Introducing and establishing weekly in-session weighing
• Introducing and adhering to a pattern of regular eating
• Thinking about addressing weight regain (if indicated)
• Involving parents to facilitate treatment
**Step two – Addressing the change**
The aim is to address weight regain and the key mechanisms that are maintaining the patient's eating disorder. The appointments are once a week for patients who are not underweight and twice a week until the rate of weight regain stabilizes in underweight patients, at which time they are held once a week. This step involves the following CBT-E modules:
• Underweight and undereating
• Overvaluation of shape and weight
• Dietary restraint
• Events and mood-related changes in eating
• Setbacks and mindsets
**Review sessions**
These are held 1 week after Step One and then every 4 weeks for:
• Collaboratively reviewing treatment compliance and progress and identifying barriers to change
• Deciding to continue with the focused form of CBT-E rather than the broad form^a^
**Step 3 – Ending well**
The aims are to ensure that progress made during treatment is maintained and the risk of relapse minimizes. There are three appointments, 2 weeks apart, covering the following:
• Addressing concerns about ending treatment
• Devising a short-term plan for addressing residual eating disorder psychopathology
• Phasing out treatment procedures, in particular self-monitoring and in-session weighing
• Devising a long-term plan for avoiding relapse
**Post-treatment review session**
Reviewing the long-term maintenance plan around 4, 12, and 20 weeks after treatment has finished

^a^The broad form of CBT-E includes four additional modules (i.e., clinical perfectionism, low self-esteem, interpersonal difficulties, and mood intolerance), one of which may be added to the focused modules in Step Two. This form of treatment is indicated if clinical perfectionism, low self-esteem, marked interpersonal difficulties, or mood intolerance are marked and appear to be maintaining the disorder and obstructing change

A detailed description of the CBT-E version for adolescents can be found in manuals for therapists [14], patients [15], and parents [16].

### Goals

The main goals of CBT-E for adolescents are the following [14]:

- Engage the young person in treatment by motivating them to recover and actively involving them in the change process.
- Help them eliminate the specific eating disorder features (e.g., preoccupation with shape, weight, and eating; dietary restraint and restriction; low weight; self-induced vomiting; laxative misuse; and excessive exercising).
- Interrupt the mechanisms maintaining these eating disorder features.
- Achieve lasting change and prevent relapse.

### Assessment and preparation

The initial interview has two main goals: (i) to assess the nature of the young person's eating problem and (ii) to engage them in the decision to begin Step One of CBT-E.

Parents are asked to allow the therapist to meet with the adolescent alone during this session. This private meeting is crucial for understanding the adolescent's perspective on their eating problem and building a solid therapeutic alliance, ensuring the therapist is not perceived as the parents' "agent. A key feature of the first CBT-E session is emphasizing that the therapist will operate entirely on the teen's behalf rather than acting at the parents' behest. Engaging the young person involves actively listening to their thoughts and concerns.

An essential strategy in the assessment/preparation phase of CBT-E is to explore with the young person whether their control over shape, weight, and eating is a choice or if it has become a problem. This is followed by formally educating the patient about the differences between the disease and the psychological models of eating disorders, along with their respective treatment implications (see Table 2).

**Table 2:** The conceptualization of eating disorders of the disease model and the psychological CBT-E model of eating disorders and their implications for the treatment

	**Disease model**	**CBT-E psychological model**
Conceptualization of eating disorders	Separates the eating disorder from the patient (externalization)	Does not separate the eating disorder from the patient
Patient Involvement	Not actively involved in the decision to change and treatment	Actively involved in the decision to change and treatment
Parents involvement	Vital (controllers)	Helpful but not essential (helpers)
Therapists	Prescribers	Collaborators—helpers

The therapist then provides a brief overview of CBT-E, asking the young person to reflect at home on what has been discussed. For homework, the therapist asks the adolescent to write down a list of the pros and cons of starting Step One of CBT-E and any questions or concerns they may have.

In the second assessment and preparation session, usually held some days later, the therapist reviews the pros and cons the adolescent has written down and addresses any issues they may have raised. The aim of this session is to encourage (rather than coerce) the young person to decide to start Step One by bolstering their interest in change and addressing any questions they may have.

### Step one – starting well and deciding to change

Step One lasts up to four weeks, during which the adolescent attends two 50-min sessions per week. In addition to fostering their engagement, the primary goal of Step One is to help the adolescent understand their eating disorder through the psychological model and to recognize the need for change (including weight regain if necessary). Additional goals include reducing their concerns about weight through the weekly collaborative weighing procedure and establishing a regular eating pattern.

By the end of Step One, the adolescent should be willing to attempt change and agree to take steps to regain weight if required. The sooner the adolescent adopts this viewpoint, the shorter Step One will be. However, if the adolescent does not agree to attempt change after eight sessions, CBT-E will be discontinued, and alternative treatments will be considered.

### Review sessions

A review session is conducted at the end of Step One for non-underweight adolescents and at flexible intervals, typically every four weeks, during Step Two for underweight adolescents. These sessions focus on assessing progress, identifying emerging barriers to change, modifying the patient's formulation as needed, and planning the following four weeks. Additionally, each review session has two key purposes:

1. Identify patients who are not doing well. It's crucial to recognize patients who are not progressing well. Achieving a positive outcome is unlikely without addressing the underlying causes of their poor response.
2. Adapt treatment. The treatment is adjusted based on the changing nature of the patient's eating disorder to ensure it remains effective and relevant. This may include the decision to use the “broad” form of CBT-E.

### Step two – addressing the change

This Step represents the main body of CBT-E. It aims to promote weight restoration (if necessary) and disrupt the main mechanisms maintaining the eating-disorder features. The way to achieve those goals varies considerably from adolescent to adolescent. To address the specific maintenance mechanisms of an individual patient, the "focused" form of CBT-E includes one or more of the following modules, as appropriate:

- Underweight and Undereating
- Body Image
- Dietary Restraint
- Events, Moods, and Eating
- Setbacks and Mindsets

For adolescents whose treatment is hindered by additional maintenance processes, the "broad" form of CBT-E is used. This version includes the following additional modules, which can be incorporated into the focused form as needed:

- Clinical Perfectionism
- Core Low Self-esteem
- Marked interpersonal Difficulties
- Mood Intolerance

### Step three – ending well

Step Three is the final stage of treatment, focusing on ending the treatment well. It consists of three appointments, each held two weeks apart. These sessions gradually shift to a future-oriented perspective, focusing less on the present. The goals of Step Three include maintaining the changes achieved during treatment and minimizing the risk of relapse. Additionally, any concerns from the adolescent and/or parent(s) about ending treatment are addressed, and specific treatment procedures (e.g., self-monitoring, in-session weighing) are gradually phased out. Toward the end of this step, the therapist collaborates with the patient to create a maintenance plan to address residual eating disorder features and prevent relapse.

### Post-treatment review sessions

These sessions occur at 4, 12, and 24-weeks post-treatment. They aim to reassess the adolescent's condition and determine the need for further intervention. Additionally, they review progress, update the long-term maintenance plan, and, if necessary (e.g., if the adolescent has not resumed having regular periods), evaluate the pros and cons of further weight gain.

### Parental involvement

Parents attend two joint sessions with the patient during the treatment's assessment and preparation phase. In the main phase of treatment, parents will need to attend one parent-only session and approximately six to ten joint sessions with the patient, which occur at the end of the individual sessions (see Table 3).

**Table 3:** The sessions involving parents in CBT-E for adolescents with eating disorders

**Assessment and preparation phase**
*• Joint patient and parent sessions* When: At the end of the individual interview with the adolescent
*•* Duration: about 15 min
• Goals: Provide the parents with information concerning their child’s eating disorder, the difference between the disease model and CBT-E psychological model of eating disorder, the nature of CBT-E, their role in the treatment, and how they can help their child to address the pros and cons of starting CBT-E
**Treatment phase**
*Parent-only session*
*•* When: First week of treatment. Other sessions can be arranged during a family crisis or extreme difficulty during mealtimes
*•* Duration: 50 min
*•* Goals: Assessing the family environment, educating the parents about their adolescent’s eating disorder and the processes maintaining it, instilling hope, addressing any self-blame, stressing the importance of creating an optimal family environment, identifying and addressing potential parental barriers to change
*Joint patient and parent sessions*
• When: At the end of individual sessions with the adolescent (four to six sessions for not-underweight patients, six to ten sessions for underweight patients)
• Duration: 15 min
• Goals: Keeping parents informed and involved in the treatment process and up to date on the patient’s progress, discussing how they might help their child to implement some essential treatment procedures

N.B. The patient should always agree with the therapist on the nature of their parent’s involvement

The first joint session with the patient during the assessment and preparation interview aims to inform family members about the nature of the eating disorder, the difference between the disease model and the psychological CBT-E model, and to provide an overview of CBT-E, focusing mainly on the roles of both parents and the patient in the treatment. The second joint interview is held after the patient's second session to inform the family about the young person's decision regarding starting Step One of the treatment.

The primary goal of the parent-only session in the first week of the treatment is to identify and address family factors that may hinder the patient's attempts to change. Discussions also include creating an optimal family environment to support the child's efforts to change. The joint sessions with patients and parents together during the treatment have two main aims: (i) To keep parents updated on the treatment process and the patient's progress, and (ii) To discuss, with the patient's prior agreement, how parents can help by creating an optimal family environment, supporting their child's attempts to change, and assisting in implementing key treatment procedures.

## Effectiveness of CBT-E for adolescents

Table 4 shows all the published studies that have evaluated the effectiveness of CBT-E for adolescents aged 12 to 19 years. The available data indicate that CBT-E is effective for adolescents with various types of eating disorders, demonstrating significant improvements in body weight, reduction of eating-disorder and general psychopathology. These positive outcomes are sustained over time, showing promise for both underweight and non-underweight adolescents and highlighting CBT-E's versatility and effectiveness in addressing the specific needs of younger patients with eating disorders. CBT-E also appears to work more effectively and quickly in adolescents than adults and achieves a similar outcome to FBT at 6- and 12-month follow-ups. Studies also indicate that CBT-E is effective for transition-age youth (ages 14 to 25) with anorexia nervosa.

**Table 4:** Studies evaluating the effectiveness of CBT-E for adolescents

First author	Year	Study design	Sample	Age	Baseline BMI-for-age percentile	Follow-up	End of treatment outcome	Follow-up outcome
Dalle Grave [17]	2013	Case series	*n* = 46; *n* = 29 completers	Mean = 15.5 years (SD = 1.3, range 13–17 years)	Mean = 2.86 (SD = 3.35)	Yes, 60 weeks (*n* = 29)	Mean BMI-for-age percentile 30.3 (SD = 16.7) 96.6% of completers had minimal residual eating-disorder psychopathology^a^	Mean BMI-for-age percentile 35.1 (SD = 26.0) 89.7% of completers had minimal residual eating-disorder psychopathology^a^
Dalle Grave [18]	2015	Case series	*n* = 68; *n* = 51 completers	Mean = 16.5 years (SD = 1.7; range 13–19 years)	Mean = 42.9 (SD = 23.2)	No	Forty-six patients (67.6%) had minimal residual eating disorder psychopathology. The frequency of binge eating, self- induced vomiting and laxative misuse decreased substantially	–-
Calugi [13]	2015	Case series	Adolescent: *N* = 46; *n* = 29 completers Adults: *n* = 49; *n* = 32 completers	Adolescents: Mean = 15.5 (SD = 1.3) Adults: Mean BMI = 24.6 (SD = 5.2)	Adolescents: Mean = 2.86 (SD = 3.35) Adults: Mean BMI = 17.7 (SD = 1.4)	No	Adolescents: 65.3% had reached a BMI-for-age percentile corresponding to BMI ≥ 18.5 in adults Adults: 36.5% reached a BMI ≥ 18.5	–-
Dalle Grave [19]	2019	Case series	*n* = 49; *n* = 35 completers	Mean = 15.5 years (SD = 1.7, range 11–18 years)	Mean = 5.67 (SD = 7.30)	Yes, 20 weeks (*n* = 29)	Mean BMI-for-age percentile 32.3 (SD = 4.6) ITT analysis Significant improvement of eating-disorder and general psychopathology	Mean BMI-for-age percentile 30.5 (SD = 2.6) ITT analysis Significant improvement of eating-disorder and general psychopathology
Dalle Grave [20]	2020	Case series	Adolescent: *N* = 74; *n* = 63 completers Adults: *n* = 81; *n* = 71 completers	Adolescents: Mean = 16.5 (SD = 1.4, range 13–19 years) Adults: Mean BMI = 30.6 (SD = 11.6, range 20–58 years)	Adolescents: Mean = 1.4 (SD = 3.2) Adults: Mean BMI = 15.1 (SD = 2.3)	Yes, 20 and 60 weeks Adolescents: *n* = 46 at 20-week and *n* = 45 at 60-week follow-up Adults: *n* = 53 at 20-week and *n* = 36 at 60-week follow-up	Adolescents: mean BMI-for-age percentile 32.1 (SE = 2.0) Adults: mean BMI 19.9 (SE = 0.1) ITT analysis Significant improvement of eating-disorder and general psychopathology for both groups	Adolescents: mean BMI-for-age percentile 31.3 (SE = 2.9) at 20-week and 23.2 (SE = 4.4) at 60-week follow-up Adults: mean BMI 19.7 (SE = 0.2) at 20-week and 18.6 (SE = 0.3) at 60-week follow-up Significant improvement of eating-disorder and general psychopathology for both groups
Le Grange [21]	2020	Non-randomized effectiveness trial	CBT-E: *n* = 46; *n* = 29 completers FBT: *n* = 51; *n* = 33 completers	Mean = 14.6 years (SD = 1.8, range 11–19)	CBT-E lower weight: % median BMI = 3.50 CBT-E higher weight: % median BMI = 11.72 FBT lower weight: % median BMI = 3.97 FBT higher weight: % median BMI = 12.09	Yes, 6 and 12 months 6 months: CBT-E *n* = 22; FBT *n* = 20 12 months: CBT-E *n* = 16; FBT *n* = 23	CBT-E lower weight: % median BMI = 94.4 Significant improvement in eating-disorder and general psychopathology CBT-E higher weight: % median BMI = 108.3 Significant improvement in eating-disorder and general psychopathology FBT lower weight: % median BMI = 96.8 Significant improvement in eating-disorder and general psychopathology FBT higher weight: % median BMI = 112.6 Significant improvement in eating-disorder and general psychopathology	CBT-E lower weight: % median BMI = 96.4 Significant improvement in eating-disorder and general psychopathology CBT-E higher weight: % median BMI = 107.2 Significant improvement in eating-disorder and general psychopathology FBT lower weight: % median BMI = 96.2 Significant improvement in eating-disorder and general psychopathology FBT higher weight: % median BMI = 111.5 Significant improvement in eating-disorder and general psychopathology
Dalle Grave [22]	2023	Case series	Adolescents: *n* = 61 *n* = 44 completers Young adults: *n* = 54 (18–25 years) *n* = 28 completers	Mean = 17 years (SD = 2.8, range 14–25)	Adolescents: Mean = 5.5 (SD = 6.3) Young adults: Mean BMI = 16.1 (SD = 1.4)		Adolescents: Mean BMI-for-age percentile 29.4 (SE = 3.6) ITT analysis Significant improvement of eating-disorder and general psychopathology Young adults: Mean BMI 19.3 (SE = 0.8) Significant improvement of eating-disorder and general psychopathology	Adolescents: Mean BMI-for-age percentile 30.0 (SE = 3.9) ITT analysis Significant improvement of eating-disorder and general psychopathology Young adults: Mean BMI 19.6 (SE = 1.3) Significant improvement of eating-disorder and general psychopathology

*BMI* Body mass index, *SD* Standard deviation, *SE* Standard error, *ITT* Intent-to-treat analysis

^a^Minimal residual eating-disorder psychopathology = global Eating Disorder Examination Questionnaire global score < 1SD above the community mean (i.e., < 2.77)

## Future challenges

A randomized control trial is essential to clarify the relative efficacy of CBT-E and FBT for the treatment of adolescent patients with eating disorders. The CogFam, a non-inferiority randomized control trial, began recruiting patients in 2024. This trial compares CBT-E and FBT in patients aged 12 to 18 with eating disorders, referred to eight outpatient clinics across four regions of Norway (Oslo, Bergen, Trondheim, and Tromsø) [23]. Key variables of interest include the relative acceptability, effectiveness, and ability of CBT-E and FBT to produce enduring change. Beyond comparing the relative effects of these treatments, it is theoretically and practically important to identify any moderators of treatment response that could guide the matching of patients to CBT-E or FBT. This consideration is plausible given the marked differences in these treatments' strategies, procedures, and proposed mechanisms of action.

It is crucial to identify the reasons for the lack of response and modify the treatment accordingly to increase the effectiveness of CBT-E for adolescents. Although this type of research is challenging, identifying mediators of CBT-E's effects will help make the treatment more effective and efficient.

Another potential strategy is to assess the relative effectiveness of the broad and focused forms of CBT-E and if the inclusion of specific modules to address comorbidities (e.g., obesity, post-traumatic stress disorder) could improve outcomes for individuals with these coexisting conditions. It is also essential to determine if and how CBT-E needs to be modified to fit the needs of individuals on the autism spectrum, with attention deficit hyperactivity disorder (ADHD), or other specific psychological features associated with eating disorders.

## Conclusions

CBT-E is a promising treatment for adolescents with eating disorders and offers several clinical advantages. It is acceptable to young people and their parents. Its collaborative nature is well-suited for ambivalent young patients who may be particularly concerned about control issues and parents who cannot participate in all treatment sessions. The transdiagnostic scope of CBT-E is another advantage, as it allows for treating the full range of disorders in adolescent patients.

## Data Availability

Not applicable.

## Abbreviations

CBT: Cognitive-behavioural therapy
CBT-E: Enhanced-cognitive behavioural therapy for eating disorders
FBT: Family-based therapy
